# Distinct and reproducible neurocognitive profiles in stable diffuse glioma: A data-driven approach to understanding cognitive heterogeneity

**DOI:** 10.1093/neuonc/noaf197

**Published:** 2025-08-27

**Authors:** Maxine Gorter, Jantine G Röttgering, Vera Belgers, Marike R van Lingen, Philip C de Witt Hamer, Linda Douw, Martin Klein

**Affiliations:** Amsterdam UMC, Vrije Universiteit Amsterdam, Anatomy and Neurosciences, Amsterdam, Netherlands; Cancer Center Amsterdam, Brain Tumor Center, Amsterdam, The Netherlands; Amsterdam UMC, Vrije Universiteit Amsterdam, Medical Psychology, Amsterdam, Netherlands; Cancer Center Amsterdam, Brain Tumor Center, Amsterdam, The Netherlands; Amsterdam UMC, Vrije Universiteit Amsterdam, Neurology, Amsterdam, Netherlands; Cancer Center Amsterdam, Brain Tumor Center, Amsterdam, The Netherlands; Amsterdam UMC, Vrije Universiteit Amsterdam, Anatomy and Neurosciences, Amsterdam, Netherlands; Amsterdam UMC, Vrije Universiteit Amsterdam, Neurosurgery, Amsterdam, Netherlands; Cancer Center Amsterdam, Brain Tumor Center, Amsterdam, The Netherlands; Amsterdam UMC, Vrije Universiteit Amsterdam, Anatomy and Neurosciences, Amsterdam, Netherlands; Cancer Center Amsterdam, Brain Tumor Center, Amsterdam, The Netherlands; Amsterdam UMC, Vrije Universiteit Amsterdam, Medical Psychology, Amsterdam, Netherlands; Cancer Center Amsterdam, Brain Tumor Center, Amsterdam, The Netherlands

**Keywords:** brain tumor, cognition, neurocognitive phenotypes, stable disease, hierarchical clustering

## Abstract

**Background:**

Glioma patients often exhibit neurocognitive deficits across multiple domains, yet studies typically assess these impairments separately. This study explores aggregated neurocognitive functioning (NCF), identifying distinct profiles and their clinical correlates.

**Methods:**

NCF in glioma patients with stable disease (≥ 2 months after treatment without clinical or radiological progression) was assessed across five domains: attention, information processing speed, verbal memory, working memory, and flexibility. We used hierarchical cluster analysis to distinguish neurocognitive profiles and replicated these profiles in an independent glioma cohort. Associations between neurocognitive profiles and clinical characteristics were examined using multinomial logistic regression.

**Results:**

Four distinct neurocognitive profiles were identified in both the study (*N* = 108) and the validation cohort (*N* = 185): a preserved, memory, processing/attention, and multi-domain profile. In both cohorts, 40% of patients exhibited impaired NCF, with deficits in at least one domain observed in 44% in the study cohort and 38% in the validation cohort. In the study cohort, tumor hemisphere and prior treatment with radiotherapy or combined radio- and chemotherapy were associated with processing/attention and multi-domain profiles. In both the cohorts, the multi-domain profile showed a weak association with self-perceived NCF. No other significant associations with patient, tumor, or treatment characteristics were observed.

**Conclusions:**

NCF in glioma patients can be classified into four reproducible neurocognitive profiles. Importantly, concurrent problems in NCF are highly prevalent. Neurocognitive profiles are associated with tumor laterality, previous oncological treatment, and self-perceived NCF, but not with other clinical characteristics.

**Key Points**

- Multi-domain neurocognitive impairment is highly prevalent in glioma.
- Neurocognitive functioning can be divided into four profiles: preserved, memory, processing/attention, and multi-domain.
- Profiles are related to tumor laterality, past treatment and self-perceived neurocognitive functioning.

**Importance of the Study**

Neurocognitive profiles provide a standardized framework for assessing neurocognitive functioning (NCF) in glioma patients, offering value in both clinical and research settings. These profiles address methodological heterogeneity and account for co-occurring deficits across multiple cognitive domains. This study is the first to examine neurocognitive profiles in glioma patients during stable disease, identifying four reproducible profiles: a preserved, memory, processing/attention, and multi-domain profile. Clinical characteristics were largely unrelated to profile classification, except for tumor laterality, past oncological treatment, and self-perceived NCF. Findings confirm that neurocognitive impairment is prevalent and rarely isolated. Implementing neurocognitive profiles enhances comparability across studies and supports individualized cognitive treatment planning.

Up to 80% of brain tumor patients experience impairment in neurocognitive functioning (NCF) at some point during the disease trajectory.^1–4^ These impairments have multifactorial causes, including tumor characteristics (eg, grade, location, molecular markers) and treatment effects (eg, surgery, radiation, chemotherapy).^5–7^ Additional factors such as anti-epileptic drugs, corticosteroids, and patient demographics (eg, functional status, age, education) also contribute to NCF.^8,9^ Studies have reported deficits across a wide range of neurocognitive domains, such as information processing speed, executive functioning (EF), episodic memory, and visuoconstructive abilities.^1,4^ However, most studies assess neurocognitive impairment using single-test or single-domain outcomes. This might be problematic because the large variety of methods to assess NCF hampers comparability of studies and consequently leading to conflicting results.^2,4,10^ Furthermore, focusing on isolated deficits overlooks the interaction between neurocognitive domains.^11–13^ Moreover, investigating neurocognition in brain tumor patients as a homogeneous group disregards individual differences in potential risk factors for neurocognitive impairment, quality of life, and rehabilitation needs. To address these challenges, we here propose the use of neurocognitive profiles as a standardized framework for assessing NCF in glioma patients.

Studies outside the neuro-oncology field have demonstrated significant advantages of the use of neurocognitive profiles over reporting single-test or single-domain outcomes. Neurocognitive profiles facilitate clinically meaningful classification of the neurocognitive status of patients,^14^ can be used to identify potential causes of neurocognitive impairment,^15,16^ track NCF after medical treatment,^17^ or predict treatment response.^18^ Concerning neuro-oncological patients, a recent study that aimed at identifying cognitive phenotypes and their trajectories in patients receiving radiotherapy, yielded three distinct neurocognitive profiles: generalized impairment, verbal memory impairment and minimal impairment.^19^ Interestingly, each of these profiles were associated with specific clinical (ie, isocitrate dehydrogenase [IDH] mutation status) and demographic characteristics (ie, educational level, gender, anxiety, and quality of life). However, a methodological drawback of the study is the use of latent profile analysis. Latent profile analysis requires an a priori specification of the number of profiles,^20^ while the neurocognitive heterogeneity in glioma is not fully understood, and therefore, the number of neurocognitive profiles often cannot be defined beforehand. Importantly, to find stable profiles, latent profile analysis requires large sample sizes,^21,22^ which is often not feasible in brain tumor patients. Additionally, this study had a heterogeneous patient population and included patients in early phases of treatment. Given the heterogeneity of neurocognitive impairment in brain tumor patients, investigating neurocognitive profiles in a more homogeneous cohort is valuable. By controlling for heterogeneity, there is a greater sensitivity to detect subtle changes, and within-group variability can be more precisely characterized. In this respect, glioma patients during their stable disease constitute a clinically relevant sample. This phase is typically characterized by the absence of anti-tumor therapy, resulting in a time without frequent hospital visits for medical procedures or checkups. During the stable disease phase, neurocognitive impairment may become more evident, significantly impacting social and professional functioning.^23,24^ Importantly, this period also presents an opportunity for cognitive rehabilitation, which can improve patients’ quality of life.

Identifying neurocognitive profiles in glioma patients provides valuable insights into the heterogeneity of NCF and its clinical relevance. However, the generalizability and robustness of such profiles remain uncertain when derived from a single cohort. Replication in an independent sample is needed to establish the stability of identified profiles across varying clinical and demographic contexts. Moreover, replication enhances the external validity of results, thereby supporting their utility for clinical stratification, personalized care, and future research aimed at linking NCF to neurobiological or treatment-related mechanisms. In line with recent calls for reproducibility in neuro-oncology research,^25^ independent validation represents a critical step toward advancing standardized neurocognitive phenotyping in glioma.

This study applied hierarchical cluster analysis to a homogeneous sample of glioma patients in the stable disease phase to identify neurocognitive profiles. To confirm reproducibility, the profiles were validated in an independent glioma cohort. Additionally, we explored the relationship between patient, tumor, and treatment characteristics and neurocognitive profiles.

## Methods

### Patients

For this observational study, two cohorts were used from the Amsterdam UMC location Vrije Universiteit Amsterdam, a tertiary referral center for neuro-oncological care in the Netherlands. From these cohorts, patients with stable disease were selected. Stable disease was defined as a period of at least two months after antitumor treatment without clinical or radiological progression.

The first cohort, the *study* cohort, included data collected between 2008 and 2021, from adult outpatients with stable disease and a histopathologically confirmed WHO grade 2-4 glioma, based on applicable WHO guidelines.^26–28^ Standardized neuropsychological assessment was obtained as part of clinical care. Approval for the use of clinical data for research purposes was granted by the Medical Ethics Review Committee Amsterdam UMC location Vrije Universiteit Amsterdam (METc VUmc 2010.126), and patients provided written informed consent.

The second cohort, the *validation* cohort, consisted of adult patients with stable disease with histopathologically confirmed low-grade gliomas (WHO grade 1 and 2)^29,30^ and was used to confirm reproducibility of our study outcomes. This cohort, collected between 1997 and 2000, was initially studied to assess the long-term effects of radiotherapy on NCF in low-grade glioma patients.^31,32^ All patients provided informed consent for using their data.

### Neurocognitive Functioning

NCF was assessed using a standardized battery of neurocognitive tests. Individual test outcomes were grouped into five neurocognitive domains (Supplementary Table S1): attention, information processing speed, verbal memory, working memory, and flexibility based on previous studies and consensus in clinical neuropsychological practice.^33–35^ Each test score was converted into a z-score using published normative data from healthy controls matched for age, sex, and education.^36–40^ Individual domain scores (see Supplementary Table S1) were computed by averaging z-scores within each domain, and impairment was defined as a z-score ≤ -1.5, a commonly used threshold as a clinically meaningful indicator of impairment.^41,42^ Both cohorts received the same battery of neuropsychological tests.

Neuropsychological assessments were conducted at least one year after diagnosis and a minimum of two months after completion of initial tumor treatment. At the time of assessment, all patients had undergone radiological evaluation confirming the absence of progression, ensuring that they were in a clinically and radiologically stable disease phase.

### Patient, Tumor, and Treatment-related Characteristics

Collected patient information include age at diagnosis, tumor grade (per WHO guidelines at the time of inclusion)^26–28^, IDH mutation status (wildtype/mutant), tumor lateralization (left/right), disease duration (months between diagnosis and assessment), prior radiation (yes/no), prior chemotherapy (yes/no), functional status (Karnofsky Performance Scale, KPS^43^; ≤ 70 / ≥ 80), and the use of anti-epileptic drugs (AED; none/mono/poly therapy).

Health-related quality of life (HRQoL) was assessed with the Medical Outcomes Study Short-Form Health Survey (SF-36)^44^ generating physical and mental component summary scores (PCS and MCS) transformed into a T-score metric as documented earlier.^45^ Higher scores of the PCS and MCS indicate better HRQoL.

Self-perceived NCF was measured using the Medical Outcomes Study Cognitive Functioning Scale (MOS-Cog).^46^ The MOS is a six-item questionnaire assessing day-to-day problems in NCF over the past month, and a higher score indicates better self-perceived NCF.

### Statistics

Hierarchical cluster analysis was used to identify neurocognitive profiles by clustering patients based on domain z-scores. Hierarchical cluster analysis is a data-driven method that aims at identifying homogeneous subgroups of patients.^47^ More specifically, with hierarchical cluster analysis, the similarity between patients’ neurocognitive domain z-scores is quantified. The algorithm uses a bottom-up approach, starting with each patient in a separate profile and combining profiles (ie, patients with similar neurocognitive profiles are merged) until all patients are grouped into a hierarchical tree (dendrogram). For combining profiles, we used Ward’s method, which analyzed the variance of profiles.^48^ This method minimizes the total within-profile variance by evaluating the sum of squared differences within each profile, thus yielding well-separated profiles. Furthermore, this method aims to preserve the pairwise distances between data points as much as possible during the clustering process. This is especially important given the type of data we used (neurocognitive scores), in which the proximity between data points provides meaningful information. Therefore, the use of domain scores, rather than test scores, is particularly useful because test scores will cluster together as they are closely related without adding information on how NCF is interrelated. The final profiles represent distinct patterns of impairments or preserved function across different neurocognitive domains. The results of the hierarchical cluster analysis, including the dendrograms were displayed in hierarchical clustered heatmaps for both cohorts.

Multinomial logistic regression was used to determine the association between clinical characteristics and neurocognitive profiles. This method, which accommodates multi-group outcomes, recoded neurocognitive profiles into binary comparisons using profile 1 (preserved NCF) as the reference. The analysis identified factors associated with belonging to profiles 2, 3, or 4 relative to profile 1. Initially, individual associations were tested (*P* < .05, see Supplementary Table S2), and significant predictors were included in the final model. Analyses were conducted in both the study and validation cohorts, though tumor grade was excluded in the validation cohort due to sample limitations. All statistical analyses were performed in R (version 4.0.3).^49^

## Results

### Patient and Tumor Characteristics

Table 1 presents clinical characteristics of the *study* (*N* = 108) and *validation* (*N* = 185) cohorts. The median age at assessment was 41 (18) years in the *study* and 40 (17) in the *validation* cohort. Sex distribution was similar (*study cohort*: 65% male, *validation cohort*: 63% male with *P *= .257 and *P *= .885, respectively). However, the cohorts differed significantly in age at diagnosis (*P *= .001), time between diagnosis and cognitive assessment (*P <* .001), tumor characteristics (location, histology, grade [all *P *< .001]), tumor treatment (*P <* .001) and the use of AEDs (*P <* .001), due to the difference in recruitment. Moreover, the *validation* cohort consists of data collected before 2000, before the implementation of molecular diagnostics, and might therefore result in outdated and unknown tumor histology (eg, oligoastrocytoma) and grade. Lastly, the two samples differed regarding their educational level (*P *= .016).

**Table 1.** Clinical Characteristics of the Patient Cohorts

	*Study cohort *	*Validation cohort*	
	*(N = 108)*	*(N = 185)*	P value*
Age in years, median (IQR)			
At diagnosis	40 (16)	35 (16)	*.001*
At assessment	41 (18)	40 (17)	.257
Months since diagnosis, median (IQR)	24 (12)	53 (50)	*<.001*
Sex, no. (%)			.885
Male	70 (65)	117 (63)	
Educational level (Verhage)**, no. (%)			*.016*
Low (1-4)	32 (30)	85 (46)	
Middle (5)	24 (22)	26 (14)	
High (6-7)	52 (48)	74 (40)	
Tumor Location, no. (%)			*<.001*
Left	56 (52)	81 (44)	
*Unknown*		22 (12)	
Histology; no. (%)			*<.001*
Astrocytoma	50 (46)	119 (64)	
Oligodendroglioma	45 (42)	38 (21)	
Glioblastoma	13 (12)	0 (0)	
*Oligoastrocytoma*		12 (6)	
*Unknown*		16 (9)	
Glioma WHO grade***, no.(%)			*<.001*
1		11 (6)	
2	70 (65)	153 (83)	
3	25 (23)		
4	13 (12)		
*Unknown*		21 (11)	
IDH mutation status and 1p/19q deletion, no.(%)			NA
IDH mutation + 1p/19q codeleted	37 (34)		
IDH mutation + 1p/19q non-codeleted	47 (44)		
IDH wild type	10 (9)		
*Not tested/Unknown*	14 (13)	185 (100)	
Tumor treatment, no.(%)			*<.001*
No radio- or chemotherapy	34 (31)	81 (44)	
Only radiotherapy	19 (18)	81 (44)	
Radio- and chemotherapy	55 (51)	2 (1)	
*Unknown*		21 (11)	
KPS, no.(%)			.11
≤ 70	13 (12)	11 (6)	
≥ 80	91 (84)	165 (89)	
*Unknown*	4 (4)	9 (5)	
Use of AEDs, no. (%)			*<.001*
None	16 (15)	52 (28)	
Monotherapy	73 (67)	72 (39)	
Poly therapy	17 (16)	56 (30)	
*Unknown*	2 (2)	5 (3)	

IQR, Inter Quartile Range; KPS, Karnofsky score; AEDs, Anti-epileptic drugs.^1.^.*Mann-Whitney U tests were used for continuous variables and Chi-square tests for categorical variables. P < .05 was condidered significant. **Education in Verhage educational classification.^50^ ***WHO classification according WHO guidelines that were applicable during inclusion.^26–30^

### Prevalence of Neurocognitive Deficits

**Figure 1.**
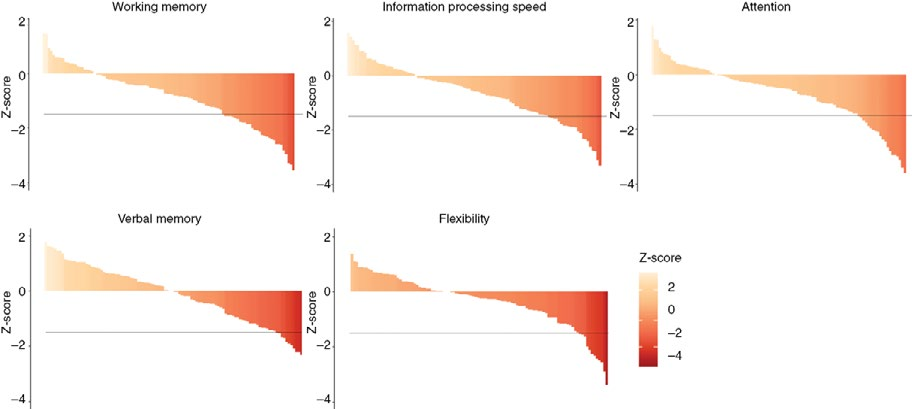
Waterfall plots showing the distribution of z-scores of neurocognitive functioning per domain of the *study* cohort. Each bar represents a patient, with darker red colors representing lower z-scores. The y-axis shows z-scores, and the horizontal line indicates the threshold of -1.5 SD below that of healthy controls.

Neurocognitive impairment in one or more domains was found in 47 patients in our *study* cohort (44%). Among these, 29 patients (62%) exhibited deficits in multiple domains. The neurocognitive domain outcomes are displayed in Figure 1. Neurocognitive domains affected in decreasing frequency were: working memory (31%), information processing speed (23%), attention (21%), verbal memory (12%), and flexibility (12%). A comparable prevalence of neurocognitive deficits was found in the *validation* cohort (see Supplementary Figure S1 and Supplementary Table S5).

### Neurocognitive Profiles

**Figure 2.**
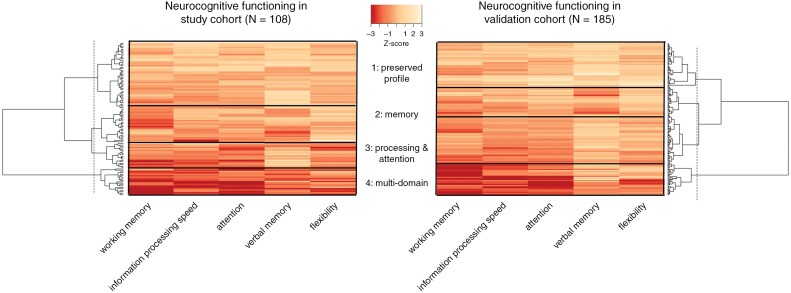
Heatmaps of neurocognitive profiles. The heatmap visualizes the arrangement of patients based on similarity, in which each row represents a patient. Darker red colors indicate lower z-scores. On the left and right sides of the heatmaps, a dendrogram is presented, which provides a visual representation of the hierarchical clustering results. The arrangement and length of the branches in the dendrogram illustrate the similarity between patients: the greater the height, the greater the differences between clusters. The dotted line is the pruning line, which thresholds the number of profiles (for both cohorts, the number of profiles is four).

A hierarchical clustered heatmap and dendrogram identified four neurocognitive profiles in both cohorts (Figure 2). By reading the dendrogram of both cohorts, the first separation of patients was made between patients with impairments in multiple domains (profile 4: multi-domain) and patients with no or a few neurocognitive impairment (profiles 1-3). The latter group included three subgroups: patients with preserved NCF (profile 1: preserved), patients with mainly decreased functioning in verbal and working memory (profile 2: memory), and patients having mainly impairment in information processing speed and/or attention (profile 3: processing/attention). Both cohorts showed a similar proportion of patients within each profile (Table 2).

**Table 2.** Validation of Profiles in an Independent Cohort

	*Cohort*		
	*Study* *(N = 108)*	*Validation* *(N = 185)*	*χ* ^*2*^ *test statistic, P-value*
*Proportion of patients per profile,* % (*n*)			
1: Preserved profile	44 % (47)	28 % (52)	5.7, *P = *.125
2: Memory profile	22 % (24)	32 % (59)	
3: Processing/attention profile	17 % (18)	19 % (35)	
4: Multi-domain profile	18 % (19)	21 % (39)	

χ^2^ = Chi-square test. The test showed no significant (*P* < .05) difference between the two cohorts.

### Relation Between Cognitive Profiles and Clinical Characteristics

Multinomial logistic regressions (Table 3) in the *study* cohort revealed significant associations between the neurocognitive profiles and tumor hemisphere, tumor treatment, and self-perceived NCF. Having a right sided tumor was associated with lower odds for being in the processing/attention and the multi-domain profiles (ie, profile 3 and 4; see Table 3) compared to being in the preserved profile (profile 1) (*β *= −1.32, *P = *.04 and *β *= −2.70, *P = *.002, respectively). When patients have had radiotherapy or a combination of radio- and chemotherapy, patients had higher odds of being in the multi-domain profile (profile 4) compared to being in the preserved profile (profile 1) (radiotherapy *β *= 3.0, *P = *.02 and combination *β *= 2.25, *P = *.046). Lastly, a higher self-perceived NCF was associated with a lower odds of being in the processing/attention profile (profile 3) compared to profile 1 (*β *= −0.04, *P = *.02). However, in the *validation* cohort, a higher self-perceived NCF was associated with higher odds for the multi-domain profile (profile 4) compared to profile 1 (*β *= 0.026, *P *< .001). Being male or female was not related to any of the profiles, neither were age at diagnosis, education, or functional status. Furthermore, there were no increased risks for having a higher grade or IDH-wildtype tumor. Similarly, disease duration and the use of AEDs did not pose a significant risk for being in one of the less favorable profiles. Lastly, HRQoL (PCS and MCS) was not related to any of the profiles (see Supplementary Table S1).

**Table 3.** Model Summary Table.

	*Profile 2:* *Memory*		*Profile 3:* *Processing/attention*		*Profile 4:* *Multi-domain*	
*Independent variables*	*Coeff. (β)*	*P-value*	*Coeff. (β)*	*P-value*	*Coeff. (β)*	*P-value*
Intercept	0.77	.49	1.95	.10	-0.67	.68
Tumor hemisphere						
Right	-0.83	.13	***-1.32***	***.04***	***-2.70***	***.002***
Treatment (radio- and/or chemotherapy)						
Combination (radio- and chemotherapy)	-0.19	.75	-0.03	.96	***2.25***	***.046***
Only radiotherapy	1.19	.13	0.79	.40	***3.00***	***.02***
Self-perceived NCF (MOS-cog score)	-0.02	.24	***-0.04***	***.02***	***-*** 0.02	.25

Multinomial logistic regression for the *study* cohort. In this model, profile 1 (cognitively preserved) is used as the reference profile. ***Bold/italic numbers*** represent significant *P*-values (*P* < .05). HRQoL, Health-related quality of life; NCF, neurocognitive functioning.

## Discussion

This study aimed at identifying reproducible neurocognitive profiles in glioma patients during stable disease. We found four distinct profiles: a preserved, memory, processing/attention, and multi-domain impairment profile. Importantly, the reproducibility of these profiles was corroborated in an independent low-grade glioma patient sample, indicating that these results are generalizable to glioma patients with stable disease. Clinical characteristics were largely unrelated to any of the profiles, except for tumor laterality, past oncological treatment, and self-perceived NCF.

The emergence of four distinct profiles suggests that it is possible to aggregate heterogenous neurocognitive measures in glioma patients into consistent and reproducible patterns. Compared to the recent study of Reyes and colleagues^19^ we found four profiles instead of three. However, the content of the three overlapping profiles is roughly the same, that is, a multi-domain, memory, and preserved profile. Importantly, the identification of profiles confirms that neurocognitive deficits rarely occur in isolation but rather co-occur. To illustrate, in our study, we showed that working memory and information processing speed were most frequently affected. However, if we would only focus on these two deficits, we would neglect the co-occurrence of more subtle deficits or sub-optimal performance in other domains. For example, many patients with working memory deficits also show a lower performance in verbal memory. Possibly, the inability to retain information for a short -time (working memory) influences the ability to recall information after a longer period (verbal memory). This insight could be valuable in the development of treatments and underscores the importance of a personalized patient-centered approach in both research as clinical practice.

Importantly, the neurocognitive profiles were related to treatment and tumor characteristics. Having had radiotherapy or combined radio- and chemotherapy was associated with a higher risk of being in the multi-domain profile, which aligns with previous literature about the effects of tumor treatment in glioma.^32,51,52^ Additionally, patients with right-sided tumors were less likely to be in the processing/attention and multi-domain profiles compared to the preserved profile, similar to previous literature and the study of Reyes and colleagues.^19,52^ However, in contrast to the study by Reyes et al, other tumor characteristics, such as IDH mutation, and patient factors, such as age at diagnosis, education, sex, functional status, and HRQoL, were not significantly associated with the identified neurocognitive profiles in our study. These different findings could be the result of different methods used for identifying profiles and relating them to clinical variables, as Reyes et al used latent profile analysis while we used multinomial logistic regression analysis. For constructing profiles, an important consideration is the handling of small sample sizes (*n* ≈ 100). Latent profile has a risk of an underpowered model with small sample size (eg, *n* < 300), while hierarchical cluster analysis can be applied to smaller datasets without concern for overfitting or convergence issues.^20,21,47^ Alternatively, the difference in associations with profiles may be explained by other differences between the study of Reyes et al. and the present study. The study population of Reyes included patients in early phases of treatment with more heterogeneity in tumor characteristics, while we had a more homogeneous sample and included only patients during the stable disease phase, potentially leading to selection bias in our study. Importantly, while the four neurocognitive profiles were replicated across two independent cohorts, the associations with clinical characteristics were modest and not always consistent. This highlights the need for further validation of the clinical and biological relevance of these profiles, particularly in cohorts with complete molecular data and longitudinal follow-up. Future studies should explore whether these profiles are predictive of functional outcomes, disease progression, or response to treatment, in order to establish their utility in clinical decision making.

The relation between objective NCF and self-perceived NCF is not straight forward. While some studies show a relation between objective measures and subjective experience of NCF, other studies found no or very weak associations.^9,53^ Similarly, our study shows mixed and weak results for the relation between the neurocognitive profiles and self-perceived cognition. While a better self-perceived NCF is related to a lower chance for being in the processing/attention profile in the study cohort, an opposite relation is found in the validation cohort (ie, better self-perceived NCF is related to a higher chance of being in the multi-domain profile). Even though the present study does not show consistent relations between the neurocognitive profiles and self-perceived NCF, it might still be important to assess both objective and subjective measures. Other studies suggest that self-perceived NCF might be more related to self-reported mental health symptoms rather than neurocognitive problems,^9,53^ which emphasizes the importance to combine objective measurement with self-report scales.

With regard to the practical implications of this study, neurocognitive profiles could be used as a framework to classify NCF of patients. This may be beneficial in research settings because neurocognitive profiles facilitate the comparison and aggregation of different studies and cohorts, as the profiles are not hindered by the heterogeneity in methods to assess NCF. This is in particular important since most glioma studies are underpowered due to small sample sizes. Furthermore, in clinical practice, the neurocognitive profiles may result in a better selection of treatment to alleviate the cognitive symptoms. For example, patients in profiles 2 (memory) and 3 (processing/attention) might require different strategies (eg, memory strategy training and metacognitive strategy training,^54^ respectively) compared to patients with generalized neurocognitive impairment (eg, some attention and intellectual awareness are needed for cognitive training). Ultimately, these profiles might be used to classify individual patients. This would contribute to patient-centered care through psycho-education for patients and caregivers, thereby enhancing awareness and managing expectations in line with their profile. To achieve this, the field can be informed by research from other neurological conditions, in which clinical frameworks are developed for cognitive profiling.^55–57^ To summarize, by using neurocognitive profiles, we can better compare outcomes from different cohorts and diseases, providing clinicians the opportunity to more systematically identify and treat their patients, and provide researchers the opportunity to develop targeted treatments.

This study evidently has its strengths and limitations. A major strength of this study is the replication of profiles in an independent glioma cohort, indicating the reproducibility of the results. Moreover, this study showed an innovative way of investigating NCF in glioma, which might pave the way to better comparison of studies, patient care, and improved neurocognitive treatments. However, a limitation of our study is that, given that the *validation* dataset predates 2000 and had different sampling methods, we could not perform all regression analyses in our *validation* cohort due to missing variables. Additionally, differences in tumor classification standards at the time of the *validation* dataset may have impacted histological and grade distributions, potentially influencing cohort comparisons. Nevertheless, in neither cohort, a relation between the neurocognitive profiles and tumor grade was found, suggesting a limited impact of these developments on our findings. On the other hand, the temporal gap between cohorts also aligns with advances in glioma treatment, which could potentially partly explain the lack of association between the oncological treatment and profiles in the validation cohort. Second, as the majority of patients in our samples had lower-grade gliomas, many of which were likely IDH-mutant, caution is warranted when generalizing these findings to broader glioma populations. In our study cohort, we did not observe an association between IDH-mutation status and neurocognitive profiles, while Reyes and colleagues did find more IDH-WT in the multi-domain profile. More studies are needed to clarify the role of molecular subtype in shaping NCF. Another limitation may be the use of a standardized neuropsychological test battery in both cohorts, which might not have captured all possible neurocognitive deficits. To illustrate, problems in visuo-constructive abilities and social cognition were not part of this battery and therefore not captured in the neurocognitive profiles. In future studies, it would be of interest to examine whether the neurocognitive profiles are consistent when using different neuropsychological test batteries and whether neurocognitive profiles of different patient populations yield the same results. Furthermore, investigating potential biological underpinnings of the neurocognitive profiles may provide insight into why some patients develop multi-domain deficits while others show preserved or domain-specific impairment. While the current study was not designed to explore the biological basis of these profiles, future research with mechanistic aims may help elucidate these pathways.

To summarize, our study found four reproducible neurocognitive profiles in glioma patients with stable disease. We showed that a substantial part of these patients have impaired NCF and that neurocognitive deficits do not occur in isolation. The neurocognitive profiles were related to tumor lateralization, past oncological treatment, and self-perceived NCF but not to other patient, tumor, or treatment factors. Importantly, these profiles have potential as a new classification framework of NCF in glioma patients, as they might be beneficial to both clinical practice and research. To move the field forward, more research on prospective neurocognitive data is needed to investigate the prognostic value of these neurocognitive profiles and examine how these profiles can be used for individual patients.

## Supplementary Material

noaf197_Supplementary_Materials_1

## Data Availability

Data will be made available upon reasonable request.
