# Supplementary material for: Distinct and reproducible neurocognitive profiles in stable diffuse glioma: A data-driven approach to understanding cognitive heterogeneity
Source: Neuro Oncol. 2025 Aug 27;27(12):3250–9. doi: 10.1093/neuonc/noaf197 (PMC12916723; doi:10.1093/neuonc/noaf197)
Supplement: noaf197_Supplementary_Materials_1 [file noaf197_supplementary_materials_1.docx]

**Supplementary Materials**

## ***Prevalence of neurocognitive deficits of the validation cohort***

**
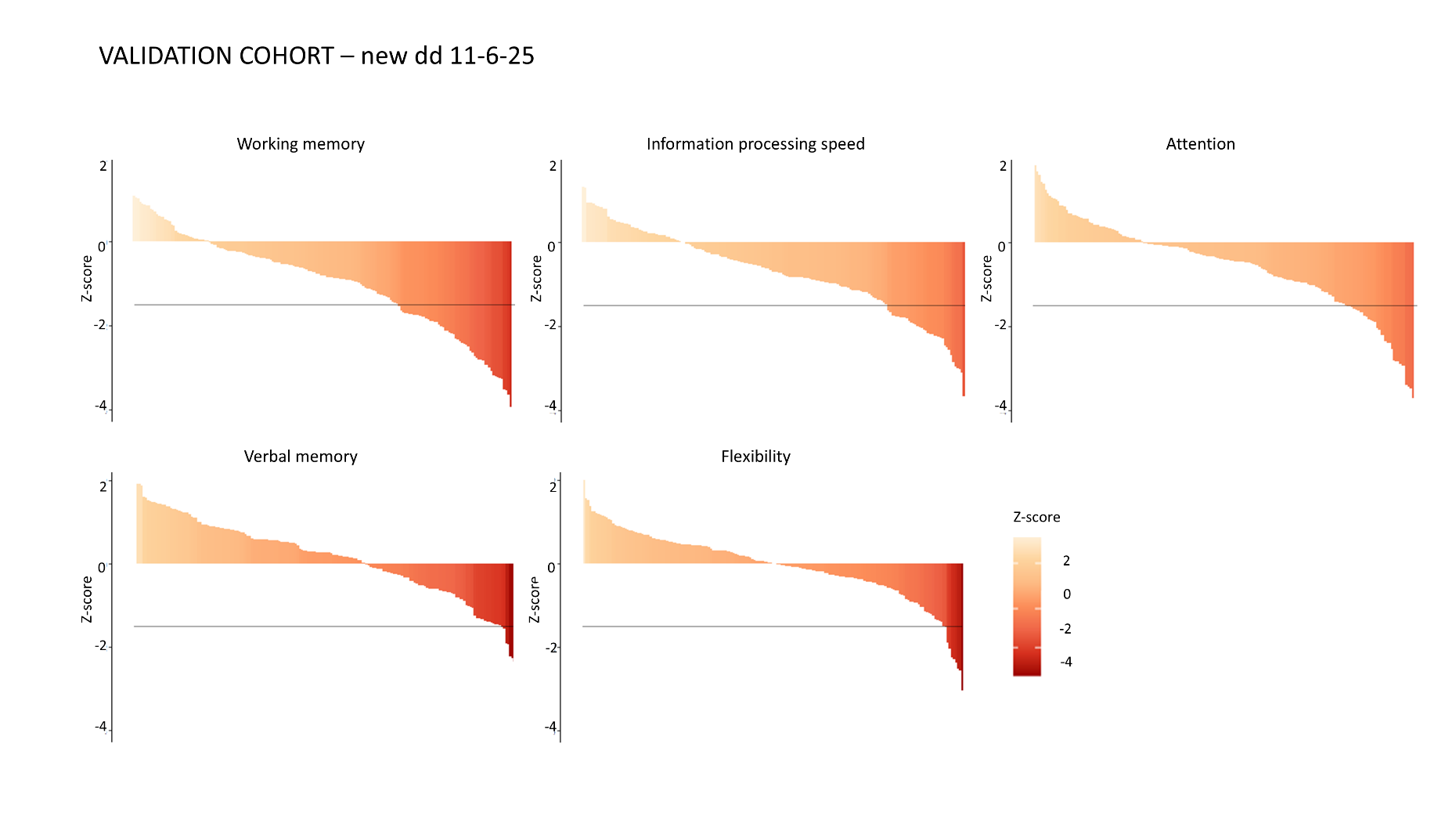
**Neurocognitive impairment in one or more domains was found in 70 patients of our validation cohort (38%). Of these neurocognitively impaired patients, 43 patients (61%) had deficits in more than one domain. The neurocognitive domain outcomes are displayed in Figure S1. Neurocognitive domains affected in decreasing frequency were: working memory (31%), information processing speed (21%), attention (19%), flexibility (5%), and verbal memory (4%).

**Figure S1**. Waterfall plots of neurocognitive data from validation cohort

**Table S1**. Tests and measures used per neurocognitive domain.

| Neurocognitive domain | Neurocognitive (sub)test | Description | Measure |
| --- | --- | --- | --- |
| Attention | Stroop Test - Card II color condition  Stroop Test - Card III color-word condition  Concept Shifting Test - Condition B letters  Concept Shifting Test - condition C number-letter | Name the colors presented on a page as quickly as possible.  Name the color of the ink in which each word is printed as quickly as possible.  Cross out the random ordered letters in alphabetical order.  Cross out the numbers and letters in chronical order, while alternating between numbers and letters. | All tests: time in seconds^b^ |
| Information processing speed | Stroop Test - Card I word condition  Concept Shifting Test - Condition A numbers  Letter Digit Substitution Test –  Writing  Letter Digit Substitution Test –  Reading | Name the words presented on a page as quickly as possible.  Cross out the random ordered numbers in chronical order.  Write down the randomized letter with the appropriate digit indicated by the key.  Name the appropriate digit with the randomized letter as indicated by the key. | Time in seconds^b^  Time in seconds^b^  Number correct (60s): 0-125^a^  Number correct (60s): 0-125)^a^ |
| Verbal memory | Auditory Verbal Learning Test – Total correct  Auditory Verbal Learning Test – Delayed Recall  Auditory Verbal Learning Test – Delayed recognition | Five learning trials of 15 words  Reproduce the 15 words after a 20-minute delay  Indicate (yes/no) the target words list of 30 words after a 20-minute delay | Number correct: 0-75)^a^  Number correct: 0-15^a^  Number correct: 0-30^a^ |
| Working memory | Memory Scanning Test – Intercept  Memory Scanning Test – Slope | Cross out 1,2,3, and 4 letters (targets they have to remember) out of a matrix of 10x12 items with 100 distractors and 20 targets. | Both tests: time score^b^ |
| Flexibility | Categorical Word Fluency Test – Category  Stroop Test – Interference score  Concept Shifting Test – Shifting score | Name as many animals as possible in 60 seconds.  Stroop Card III-corrected for card I.  Condition C corrected for Condition A and B. | Number correct: 0 - ∞^a^  Time score^b^  Time score^b^ |

^a^ Higher score means better functioning, ^b^ Higher score means worse functioning

**Table S2.** Multinomial logistic regressions of all clinical variables in the test cohort.

|  | | | Profile 2 | | Profile 3 | | Profile 4 | |
| --- | --- | --- | --- | --- | --- | --- | --- | --- |
| *Independent variables* | | | *Coeff. (β)* | *p-value* | *Coeff. (β)* | *p-value* | *Coeff. (β)* | *p-value* |
| ***Patient characteristics*** | | | | | | | | |
| Intercept | | | -1.77 | 0.07 | -0.72 | 0.49 | -1.85 | 0.08 |
| Age at diagnosis | | | 0.03 | 0.23 | -0.06 | 0.82 | 0.02 | 0.34 |
| Intercept | | | -0.93 | 0.004 | -1.19 | 0.001 | -0.86 | 0.007 |
| Sex (female) | | | 0.69 | 0.18 | 0.63 | 0.27 | -0.17 | 0.78 |
| Intercept | | | -1.22 | 0.02 | -1.22 | 0.02 | -1.22 | 0.02 |
| Education | | |  |  |  |  |  |  |
|  | Middle | | 0.13 | 0.88 | 0.13 | 0.88 | 1.22 | 0.08 |
|  | High | | 0.95 | 0.12 | 0.48 | 0.45 | -0.21 | 0.77 |
| Intercept | | | 0.51 | 0.48 | 0.001 | 1.0 | -0.4 | 0.66 |
| KPS | | |  |  |  |  |  |  |
|  | ≥ 80 | | -1.44 | 0.07 | -1.05 | 0.23 | -0.58 | 0.54 |
| ***Tumor characteristics*** | | | | | | | | |
| Intercept | | | -0.79 | 0.01 | -1.01 | 0.003 | -1.19 | 0.001 |
| Grade | | |  |  |  |  |  |  |
|  | Grade 3 | | 0.94 | 0.14 | 1.01 | 0.13 | 1.19 | 0.08 |
|  | Grade 4 | | -0.60 | 0.48 | -9.80 | 0.90 | 0.21 | 0.78 |
| Intercept | | | -0.92 | 0.007 | -1.10 | 0.002 | -1.46 | 0.001 |
| IDH-status | | |  |  |  |  |  |  |
|  | IDH WT | | 1.61 | 0.21 | 1.79 | 0.16 | 1.46 | 0.32 |
| Intercept | | | -8.47 | 0.90 | 0.001 | 1.0 | 0.69 | 0.57 |
| Side | | |  |  |  |  |  |  |
|  | | Left | 8.20 | 0.91 | -0.44 | 0.77 | -0.81 | 0.52 |
|  | | Right | 7.50 | 0.91 | -1.58 | 0.29 | ***3.36*** | ***0.02*** |
| Intercept | | | -0.91 | 0.02 | -0.76 | 0.09 | -1.49 | <0.001 |
| Disease duration | | | 0.01 | 0.40 | -0.01 | 0.59 | 0.02 | 0.06 |
| ***Treatment options*** | | | | | | | | |
| Intercept | | | -0.81 | 0.06 | -0.94 | 0.03 | -2.89 | 0.005 |
| Treatments | | |  |  |  |  |  |  |
|  | RT & XT | | -0.06 | 0.91 | -0.15 | 0.80 | ***2.28*** | ***0.04*** |
|  | Only RT | | 0.99 | 0.18 | 0.43 | 0.61 | ***2.89*** | ***0.02*** |
| Intercept | | | -0.66 | 0.03 | -0.95 | 0.005 | -0.79 | 0.01 |
| AEDs | | |  |  |  |  |  |  |
|  | None | | 0.65 | 0.77 | 108 | 0.92 | 0.75 | 0.80 |
|  | Polytherapy | | 1.10 | 0.20 | 0.64 | 0.30 | 0.85 | 0.49 |
| ***QoL & subjective NCF*** | | | | | | | | |
| Intercept | | | -1.68 | 0.63 | 0.20 | 0.96 | -8.22 | 0.06 |
| HR-QoL (SF-36) | | |  |  |  |  |  |  |
|  | | PCS | -0.01 | 0.70 | -0.04 | 0.12 | -0.002 | 0.93 |
|  | | MCS | 0.03 | 0.65 | 0.01 | 0.86 | 0.15 | 0.07 |
| Intercept | | | 0.24 | 0.80 | 1.12 | 0.25 | 0.27 | 0.79 |
| Subjective NCF (MOS-COG) | | | -0.01 | 0.31 | ***-0.03*** | ***0.03*** | -0.02 | 0.22 |

***Bold/italic numbers*** represent significant p-values (*p* <0.05). IDH WT, IDH wild type; RT, radiotherapy; XT, chemotherapy; AEDs, Anti-epileptic drugs; HR-QoL, Health-related quality of life; PCS, physical component scale; MCS, mental component scale; NCF, neurocognitive functioning

**Table S3.** Model summary table for the validation cohort.

|  |  | *Profile 2* | | *Profile 3* | | *Profile 4* | |
| --- | --- | --- | --- | --- | --- | --- | --- |
| *Independent variables* | | *Coeff. (β)* | *p-value* | *Coeff. (β)* | *p-value* | *Coeff. (β)* | *p-value* |
| Intercept | | -0.94 | 0.10 | -0.59 | 0.22 | -1.87 | 0.002 |
| Tumor hemisphere | |  |  |  |  |  |  |
|  | Right | -010 | 0.86 | -0.32 | 0.45 | -0.48 | 0.33 |
| Treatment (radio- and/or chemotherapy) | |  |  |  |  |  |  |
|  | Only radiotherapy | 0.74 | 0.14 | 0.80 | 0.06 | 0.16 | 0.75 |
| Self-perceived NCF (MOS-cog score) | | 0.002 | 0.86 | 0.02 | 0.20 | ***0.026*** | ***<0.001*** |

Multinomial logistic regression for the validation cohort. In this model, profile 1 (cognitively preserved) is used as the reference profile. ***Bold/italic numbers*** represent significant p-values (*p* <0.05). HR-QoL, Health-related quality of life; MCS, mental component score.

**Table S4**. Clinical characteristics of the study cohort per profile.

|  | |  | *Cognitive profiles* | | | | |
| --- | --- | --- | --- | --- | --- | --- | --- |
|  | | *All patients (N=108)* | | *Profile 1*  *(N=47)* | *Profile 2 (N=24)* | *Profile 3 (N=18)* | *Profile 4 (N=19)* |
| Age at testing, mean (years) ± SD | | 43 ± 12 | | 41 ± 11 | 45 ± 11 | 40 ± 10 | 45 ± 15 |
| Sex, no. (%) | |  | |  |  |  |  |
|  | Male | 70 (65) | | 33 (70) | 13 (54) | 10 (56) | 14 (74) |
| Educational level^*^, median ± IQR | | 5 ± 2 | | 5 ± 2 | 6 ± 2 | 6 ± 3 | 5 ± 1 |
| Tumor location; left, no. (%) | | 56 (52) | | 17 (36) | 13 (54) | 11 (61) | 15 (79) |
| Histology; no. (%) | |  | |  |  |  |  |
|  | Astrocytoma | 50 (46) | | 22 (46) | 12 (50) | 12 (67) | 4 (21) |
|  | Oligodendroglioma | 45 (42) | | 17 (15) | 10 (42) | 6 (33) | 12 (63) |
|  | Glioblastoma | 13 (12) | | 8 (17) | 2 (8) | 0 (0) | 3 (16) |
| Glioma WHO grade, no.(%) | |  | |  |  |  |  |
|  | 2 | 70 (65) | | 33 (70) | 15 (63) | 12 (67) | 10 (53) |
|  | 3 | 25 (23) | | 6 (13) | 7 (29) | 6 (33) | 6 (32) |
|  | 4 | 13 (12) | | 8 (17) | 2 (8) | 0 (0) | 3 (16) |
| IDH mutation status and 1p/19q deletion, no.(%) | |  | |  |  |  |  |
|  | IDH mutation + 1p/19q codeletion | 37 (34) | | 12 (26) | 10 (42) | 4 (22) | 11 (58) |
|  | IDH mutation + 1p/19q non-codeletion | 47 (44) | | 22 (47) | 11 (46) | 10 (56) | 4 (21) |
|  | IDH wild type | 10 (9) | | 4 (9) | 2 (8) | 1 (6) | 3 (16) |
|  | Not tested | 14 (13) | | 9 (0) | 1 (4) | 3 (17) | 1 (14) |
| KPS, median ± IQR | | 90 ± 20 | | 90 ± 20 | 85 ± 20 | 85 ± 20 | 80 ± 10 |
| Epilepsy, yes. (%) | | 90 (83) | | 39 (83) | 17 (71) | 17 (94) | 17 (89) |
| AEDs, yes. (%) | | 90 (83) | | 39 (83) | 17 (71) | 17 (94) | 17 (89) |

*Verhage educational level. KPS, Karnofsky performance scale; AED, anti-epileptic drugs.

|  | *Study cohort* | | *Validation cohort* | |
| --- | --- | --- | --- | --- |
|  | *Mean (sd)* | *% of patients with z ≤ - 1.5* | *Mean (sd)* | *% of patients with z ≤ - 1.5* |
| Attention | -0.82 (1.3) | 21 | -0.63 (1.4) | 19 |
| Information processing speed | -0.77 (1.2) | 23 | -0.86 (1.3) | 21 |
| Verbal memory | -0.18 (1.1) | 12 | 0.15 (1.0) | 4 |
| Working memory | -1.0 (1.3) | 31 | -1.1 (1.3) | 31 |
| Flexibility | -0.35 (1.0) | 12 | -0.07 (1.4) | 5 |

**Table S5**. Average domain scores and percentage of patients with suboptimal performance.
